# Neuro-Psychiatric Disorders: From Diagnosis to Care

**DOI:** 10.3390/diseases7030048

**Published:** 2019-07-05

**Authors:** Omar Cauli

**Affiliations:** Department of Nursing, University of Valencia, 46010 Valencia, Spain; Omar.Cauli@uv.es; Tel.: +34-96-386-41-82; Fax: +34-96-398-30-35

Neuro-psychiatric disorders are an important cause of poor quality of life, disability, and premature mortality. Globally, the burden of these disorders has increased substantially over the past 25 years, due to expanding population numbers and aging, despite substantial decreases in mortality rates. The pathophysiological bases of these disorders show continuous challenges, and different aspects within the same disorder appear quickly in the field of biomedical sciences. The complexity of these disorders needs the expertise of a multidisciplinary team to provide the best healthcare for these patients.

This Special Issue wishes to shed new light on this exciting and insightful field of research from a multidisciplinary perspective. This Special Issue, entitled “Neuro-Psychiatric Disorders: From Diagnosis to Care”, reflects the interplay between neurological and psychiatric sciences, with other health sciences at the leading edge of this growing research field, such as nursing science, which intensively suggest new opportunities for improving diagnosis and care, or to prevent adverse outcomes. In the Special Issue, the readership will find relevant research carried out by several health care professionals and researchers with extensive knowledge on clinical settings, and it is intended to address new issues of interest of specific importance to research and clinical practice. This Special Issue of *Diseases*, entitled “Neuro-Psychiatric Disorders: From Diagnosis to Care”, includes 11 manuscripts—specifically, 5 reviews and 6 original articles. We hope that, in reading these articles, you encounter research strategies that resonate with you and motivate you to continue to raise the bar for interdisciplinary research in neuro-psychiatric disorders.

Prado-Olivares and Chover-Sierra [1] reported that the level of preoperatory anxiety in patients undergoing cardiac surgery is significantly associated with level of studies, first surgical intervention, and the rating given to their previous surgical experience. The relationship between the received information and the anxiety level is inversely correlated, so these patients should be provided with all the information through an individualized intervention to reduce their anxiety and its related adverse outcomes. Chalimourdas and co-workers [2] analyzed the psychometric properties, factor structure, and evidence for measurement invariance for the disgust scale applied to the Greek population. Demographic influences on the responses were present, especially the significant impact of gender. In clinical practice, the scale can be used to get more insight into the symptoms, in several psychopathological conditions, that have been shown to be associated with disgust (e.g., anxiety- and obsessive-compulsive disorder-related disorders). Lavdaniti and co-workers [3] assessed the factors influencing quality of life (QoL) in patients with breast cancer six months after the completion of adjuvant chemotherapy. Age, menopausal status, and previous therapy were significantly associated to differences in QoL. These results suggest that health care professionals should be aware of the factors that influence the different domains (physical role, bodily pain, vitality) of QoL in order to meet patients’ needs following acute treatment. Morio and co-workers [4] analyzed the factors that influence falls in elderly people and found that individuals who experienced falls had a shortened step length, large fluctuations in their pace, and a slow walking speed. Authors identified knee extension muscle force and step length-to-height ratio as factors significantly related to a better walking speed. Vlachou and co-workers [5] studied for the first time the prevalence, characteristics, and impact of dysmenorrhea on the wellbeing (exercising, and social and academic functioning) of university students in Greece. The prevalence of dysmenorrhea was as high as 89.2% and the rate of severe intensity was 52.5%. Factors that were significantly associated with severe dysmenorrhea were family history, early menarche, and menstruation duration. Finally, university activities affected by students with severe pain included class attendance, personal studying, exercising, and socializing. Di Iorio and co-workers [6] investigated the role of action observation (AO) in improving balance and gait and in reducing falls in individuals with Parkinson’s disease (PD). AO significantly improved some symptoms and outcomes such as motor symptoms, mental score, freezing of gait, and the event-related potential P300. AO shows its effectiveness in learning or enhancing the quality of execution of specific motor skills, and it is a safe and feasible paradigm of rehabilitative exercise in cognitively preserved PD patients. Hiriscau and Bodolea [7] reviewed the impact of depression and anxiety, and their effects on clinical outcomes and prognosis in frail patients with heart failure (HF). Depression represents an independent risk factor of cardiac-related incidents and death, and a strong predictor of rehospitalization. Anxiety seems to be an adequate predictor only in conjunction with depression. The care of HF patients should include a proper control of depressive and anxiety symptoms in order to reduce the morbidity and mortality rates. Matsuda and co-workers [8] summarized the roles of the PI3K/AKT/GSK3 pathway involved in psychiatric disorders. The manuscript provides novel insights into the mechanism of mental disorder involved in psychiatric illnesses and opens future opportunities for new targets for diagnostic and/or therapeutic procedures in these disorders. Brognara and co-workers [9] reviewed the available literature concerning the application of wearable sensors to assess spatio-temporal parameters of gait in patients with PD. Wearable motion sensors are useful, non-invasive, low-cost, and objective tools to analyze gait impairment in PD patients. These sensors could help clinicians to diagnose and monitor the progression of PD patients and to evaluate the clinical efficacy of therapeutic interventions. Pérez-Ros and Martínez-Arnau [10] analyzed the tools for the assessment of delirium in older individuals presenting at emergency departments. The presence of dementia in the assessment of delirium may induce sensitivity bias. Despite the existence of numerous delirium rating scales, scales taking less than three minutes to complete are recommended. The scale affording the highest sensitivity and specificity in older people with and without dementia is the Four “A”s Test and, moreover, it does not require training on the part of the rater, and can be performed in under two minutes. Gómez-Rubio and Trapero [11] examined the current evidence about the beneficial effect of physical exercise on the immune system in patients with schizophrenia. Authors specifically analyzed the interleukin-6 (IL-6) pathway as a potential mechanism resulting in these positive effects. Inflammation and high levels of IL-6 are associated with both the severity of schizophrenia and the cognitive impairment suffered throughout the disease. Performing regular exercise can modulate IL-6 by lowering its basal levels and by causing lower acute increases in the plasma levels of this cytokine in response to exercise (an anti-inflammatory response to physical exertion). In concluding the Editorial, I would like to thank the editors of *Diseases* and the MPDI Editorial staff for offering me the opportunity to serve as guest editor for this Special Issue. I greatly appreciated the work of all the contributors and their support for this Special Issue leading to a rewarding experience in both my professional and personal life.
